# Genetic Workup for Charcot–Marie–Tooth Neuropathy: A Retrospective Single-Site Experience Covering 15 Years

**DOI:** 10.3390/life12030402

**Published:** 2022-03-10

**Authors:** Chiara Gemelli, Alessandro Geroldi, Sara Massucco, Lucia Trevisan, Ilaria Callegari, Lucio Marinelli, Giulia Ursino, Mehrnaz Hamedani, Giulia Mennella, Silvia Stara, Giovanni Maggi, Laura Mori, Cristina Schenone, Fabio Gotta, Serena Patrone, Alessia Mammi, Paola Origone, Valeria Prada, Lucilla Nobbio, Paola Mandich, Angelo Schenone, Emilia Bellone, Marina Grandis

**Affiliations:** 1 Department of Neurosciences, Rehabilitation, Ophthalmology, Genetic and Maternal and Infantile Sciences (DINOGMI), University of Genova, 16126 Genova, Italy; ageroldi@hotmail.com (A.G.); massucco.sara@gmail.com (S.M.); lucy.a.trevisan@gmail.com (L.T.); lucio.marinelli@unige.it (L.M.); mehrnaz_m1364@yahoo.com (M.H.); giumennella1994@libero.it (G.M.); silvia.stara@unige.it (S.S.); laura.mori@unige.it (L.M.); crischenone92@gmail.com (C.S.); serena87s@libero.i (S.P.); origone@unige.it (P.O.); valeria.prada@gmail.com (V.P.); lnobbio@neurologia.unige.it (L.N.); paola.mandich@unige.it (P.M.); aschenone@neurologia.unige.it (A.S.); emilia.bellone@unige.it (E.B.); mgrandis@neurologia.unige.it (M.G.); 2 IRCCS Ospedale Policlinico San Martino, 16132 Genoa, Italy; giovanni.maggi@hsanmartino.it (G.M.); fabio.gotta@hsanmartino.it (F.G.); alessia.cogne@gmail.com (A.M.); 3 Department of Biomedicine, University Hospital Basel, University of Basel, 4001 Basel, Switzerland; ilaria.callegari01@universitadipavia.it; 4 Unit of Neurology, ASL3 Villa Scassi Hospital, 16149 Genoa, Italy; giusi.ursino@hotmail.it; 5 FISM, Fondazione Italiana Sclerosi Multipla, 16126 Genoa, Italy

**Keywords:** Charcot–Marie–Tooth (CMT) disease, neuropathy, genetic, phenotype

## Abstract

Charcot–Marie–Tooth (CMT) disease is the most commonly inherited neurological disorder. This study includes patients affected by CMT during regular follow-ups at the CMT clinic in Genova, a neuromuscular university center in the northwest of Italy, with the aim of describing the genetic distribution of CMT subtypes in our cohort and reporting a peculiar phenotype. Since 2004, 585 patients (447 index cases) have been evaluated at our center, 64.9% of whom have a demyelinating neuropathy and 35.1% of whom have an axonal neuropathy. A genetic diagnosis was achieved in 66% of all patients, with the following distribution: CMT1A (48%), HNPP (14%), CMT1X (13%), CMT2A (5%), and P0-related neuropathies (7%), accounting all together for 87% of all the molecularly defined neuropathies. Interestingly, we observe a peculiar phenotype with initial exclusive lower limb involvement as well as lower limb involvement that is maintained over time, which we have defined as a “strictly length-dependent” phenotype. Most patients with this clinical presentation shared variants in either *HSPB1* or *MPZ* genes. The identification of distinctive phenotypes such as this one may help to address genetic diagnosis. In conclusion, we describe our diagnostic experiences as a multidisciplinary outpatient clinic, combining a gene-by-gene approach or targeted gene panels based on clinical presentation.

## 1. Introduction

Charcot–Marie–Tooth disease (CMT) is the most commonly inherited neuromuscular disorder, with a prevalence ranging from 9.7/100,000 in Serbia to 82.3/100,000 in Norway [1].

CMT comprises a group of inherited motor and sensory neuropathies that are phenotypically and genetically heterogeneous, with more than 100 different disease-associated genes identified [2].

Electrophysiological and neuropathological findings differentiate CMT forms into the demyelinating type, with motor nerve conduction velocities (mNCV) of <38 m/s from the ulnar or median nerve, and the axonal type, with an mNCV of >38 m/s [3]. This classification, somehow “didactic”, still helps to address genetic investigations or the interpretation of molecular results.

Genetic diagnosis in CMT has evolved rapidly in recent years with the introduction of next-generation sequencing (NGS) into routine diagnostic practice [4]. Since the frequencies of gene pathogenic variants may vary considerably between different populations, data on patient cohorts from different countries are useful for improving the diagnostic molecular algorithms [5].

Here we describe the clinical features as well as the distribution of genetic variants in patients evaluated at our neuromuscular center. The data presented here provide an overview of the frequencies of genetic subtypes of CMT patients in a neuromuscular center from northern Italy. Moreover, we describe a peculiar phenotype with the lower limbs predominantly involved. 

## 2. Materials and Methods

All patients evaluated at the neuromuscular center at the University of Genova between 2004 and 2020 were enrolled in this study. We selected patients affected by CMT based on the following: 

(a) The presence of a clinical motor-sensory neuropathy with or without positive family history;

(b) A neurological and neurophysiological examination demonstrating peripheral neuropathy; 

(c) The exclusion of primary acquired causes, such as inflammatory, toxic, metabolic, and infectious neuropathies. Patients carrying *TTR* pathogenic variants were also excluded. 

Pure motor or sensitive neuropathies were included as distal hereditary neuropathies. 

Patients were evaluated in an outpatient setting with a multidisciplinary evaluation from a team including a neurologist, neurophysiologist, medical geneticist, and physical medicine and rehabilitation (PM&R) physician. Our integrated approach takes into account the complexity of CMT, for which a multidisciplinary approach improves long-term care [6].

All patients on the same day were evaluated with an electrodiagnostic test in order to confirm the suspicion of hereditary neuropathy and categorize it. Patients were classified as CMT1 (demyelinating form) with a median mNCV below 38 m/s and CMT2 (axonal form) with a median mNCV above 38 m/s. Occasionally, clinical, electrophysiological, and pathological features could not fit into this classification so a third group of CMT called “intermediate CMT” was identified [3,7]. This group presented a combination of axonal and demyelinating changes reflected in electrophysiological studies with a median mNCV different from CMT1 (usually <25 m/s) and CMT2 (usually >45 m/s). This phenotype was described for different genes with X-linked transmission, such as males with *GJB1* pathogenic variants or autosomal dominant or recessive transmission. This is a complex characterization that requires a specific electrophysiological protocol [3] that was not conducted every time. In order to avoid errors, we prefer to simplify the classification using only axonal and demyelinating forms. Nerve conduction studies were also performed as a follow-up screening and to compute the CMT neuropathy score (CMTNS version 1 or 2) [8,9]. The CMTNS and CMT examination scores (CMTES version 1 or 2) were used to categorize cases into mild (CMTNS 0 to 10 or CMTES 0 to 7), moderate (CMTNS 11 to 20 or CMTES 8 to 16), and severe (CMTNS 21 to 36 or CMTES 17 to 28) [8,9].

A neurologist and medical geneticist evaluated family history, clinical and neurophysiological features and planned the diagnostic flow chart recommended for the patients. The medical geneticist helped the patients understand the clinical, ethic, technical, and familial implications involved with the genetic tests. The neurologist offered the management of supportive therapies [10]. Moreover, every two months, complex cases were discussed in a multidisciplinary team to decide the genetic workout. The PM&R physician’s assessment was performed with the help of the orthopedic technician and physiotherapist in order to customize physical therapies, orthotics, and prosthetics, and recommendations regarding exercise.

All the patients signed informed consent waivers in accordance with national laws and guidelines for genetic testing that are used in current clinical practice. This consent provides for the anonymous use of data for research and publication. Molecular analyses were performed at the Laboratory of Medical Genetics Unit, University of Genoa where genomic DNA was extracted from peripheral blood according to standard protocols.

Over the course of 15 years, different labs’ approaches and technologies have been used to achieve the molecular definition.

The presence of the 17p11.2 duplication or deletion was excluded by multiplex ligation-dependent probe amplification (MLPA) and quantitative real-time PCR (qPCR).

Initially, subsequent genetic tests were performed using a gene-by-gene approach based on clinical and electrophysiological features suggesting specific genetic defects. Most of these analyses were performed through conventional Sanger sequencing. More recently, the gene-by-gene approach has been progressively replaced by next-generation sequencing analysis.

Direct sequencing was achieved on an ABI PRISM 3130XL Genetic Analyzer (Applied Biosystems, Thermo Fisher Scientific, Waltham, MA, USA). Alignment of reference sequences and analysis were performed using the SeqScape Software version 2.7 (Thermo Fisher Scientific).

For next-generation sequencing (NGS) studies, a 56 CMT custom AmpliSeq gene panel (Thermo Fisher Scientific) (full list available on request) was run on an Ion S5 GeneStudio (Thermo Fisher Scientific) sequencer and Ion Reporter version 5.16 (Thermo Fisher Scientific), and the ANNOVAR [11] software was used for data analysis.

## 3. Results

In total, 585 patients (447 index cases; 99 familial and 348 isolated cases) were evaluated in our neuromuscular center since 2004 and received a diagnosis of CMT according to clinical and neurophysiological features. The overall mean age of our patients was 53 years (±16), and the median age was 53 years, with an age range of 13–94 years. The disease was nearly equally distributed between males and females (47% female, 53% male). Neurophysiology was consistent with a demyelinating phenotype in 290 patients (64.9%) and an axonal phenotype in 157 patients (35.1%). 

### 3.1. Genetically Confirmed Patients

Among the 585 patients, a genetic diagnosis was achieved in 391 patients (277 index cases, 79 familial and 198 isolated cases). The statistical analysis was based on index cases. Most patients were affected by demyelinating neuropathy (86%), whereas axonal forms accounted for 14% of genetically identified cases. As already described in the literature, demyelinating cases achieved a positive genetic diagnosis more frequently than axonal and intermediate ones [3,6].

In familial cases, autosomal dominant inheritance was the most frequent pattern of inheritance, accounting for 82% of cases. An X-linked inheritance was present in 14% of cases, and only a small percentage (4%) was characterized by a recessive inheritance. 

**Figure 1:**
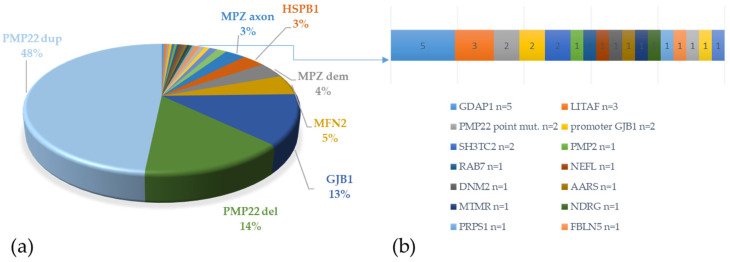
Genetic spectrum of 277 cases with pathogenic variants. (**a**) The following genes are indicated: *PMP22* dup (*n* = 134); *PMP22* del (*n* = 40); *GJB1* (*n* = 35); *MFN2* (*n* = 14); *MPZ* causing demyelinating neuropathy (*n* = 11); *MPZ* causing axonal neuropathy (*n* = 8); *HSPB1* (*n* = 9). (**b**) Other less frequent genes.

The most common genetic diagnoses were CMT1A caused by *PMP22* duplication, accounting for one half of all patients (48%); HNPP caused by *PMP22* deletion (14%); CMT1X caused by pathogenic variants in *GJB1* (13%); P0-related neuropathies caused by *MPZ* pathogenic variants (7%); CMT2A due to *MFN2* pathogenic variants (5%). All together, these accounted for 87% of all molecularly defined neuropathies. Pathogenic variants in rarely mutated genes (*SH3TC2*, *LITAF*, *RAB7A*, *NEFL*, *AARS*, *MTMR2*, *NDRG1*, *PRPS1*, *INF2*, *PMP2*, *DNM2*, *FBLN5*, *HINT1*, *IGHMBP2*, *PMP22*) each accounted for less than 1% of the total, except for *HSPB1* pathogenic variants that were found in 3% of all index cases and *GDAP1*, which accounted for 2% of all index cases. Figure 1 describes the genetic distribution of our cohort. 

NGS genetic analysis was performed in a total of 44 patients and in 10 of them, we achieved a diagnosis (two *GJB1*, one *AARS*, one *PRPS1*, one *NDRG1*, one *DNM2*, one *LITAF*, one *MFN2*, one *HINT1*, and one *FBLN5*). For seven of them, a family history of neuropathy was known.

The remaining 34 patients did not receive a genetic diagnosis despite NGS analysis.

### 3.2. Patients without Genetic Confirmation

For 194 patients, it was not possible to achieve a genetic diagnosis (33% of the whole population). Of these, index cases were 170 (20 familial and 150 isolated cases) and they more frequently presented with axonal neuropathy (69%).

Since our database includes patients evaluated during a wide time span, different genetic approaches have followed one another. This implies that most of the undiagnosed cases have been studied with a gene-by-gene approach, with a mean of four genes studied for each patient (minimum one, and maximum nine) on the basis of diagnostic algorithms. In 78 patients, three or fewer genes were analyzed. The most studied genes, after *PMP22* duplication or deletion, were *MPZ*, *HSPB1*, *GDAP1*, and *MFN2*. *MPZ* was analyzed in 68.6% of patients, followed by *HSPB1* (60.4%), *GDAP1* (55.2%), and *MFN2* (46.3%). An NGS analysis was performed on only 34 patients.

### 3.3. Genotype–Phenotype Correlation: A New CMT Phenotype

Our population displayed a relatively high frequency of pathogenic variants in the *MPZ* and *HSPB1* genes. We identified fifteen patients (nine index cases and six relatives) affected by axonal CMT associated with the *MPZ* pathogenic variant (CMT2I/2J) and nine patients (all isolated cases) affected by CMT (CMT2F, *n* = three patients) or distal hereditary motor neuropathies (dHMN), (*n* = six patients). A clearly length-dependent phenotype with exclusive involvement of the lower limbs in the earlier stage was found in 60% of cases with a pathogenic variant in *HSPB1* (five out of nine patients) and 80% of patients with the late-onset *MPZ* pathogenic variant (CMT2) (12 out of 15 patients). A complete electrophysiological study was not available for all patients. From evaluating the electrophysiological studies of patients with a clinical length-dependent phenotype, we confirmed a neuropathy confined to the lower limbs in almost 50% of *MPZ* and *HSPB1* pathogenic variants (five out of nine patients and two out of three patients, respectively) despite a long history of illness (the median number of years between the onset of neuropathy and the first evaluation with an electrophysiological study was 14; the minimum number was 7 and the maximum was 19), identifying a phenotype that was maintained over time. The age of onset was 43 ± 14 (minimum 14, maximum 64) for *MPZ* and 40 ± 20 for *HSPB1* patients (minimum 10, maximum 65). If we excluded patients with an onset before the age of 40, the percentage of patients with length-dependent phenotypes rose to 83% in *HSPB1* patients and remained elevated (73%) for *MPZ*. This result could be associated with the lower illness duration in patients with adult onset at the time of the first evaluation in our center. Summarizing, this exclusive involvement of the lower limbs was the first sign of the disease and was maintained over time, as demonstrated by the long history of illness in our patients. A later progression with the extension of neuropathy in the upper limbs was nevertheless present.

None of the patients showed severe neuropathy. Patients with the *MPZ* pathogenic variant presented a mild phenotype in 78% (11 out of 14 patients), with a mean of 14.2 years of illness duration. The percentage decreased to 66% (six out of nine patients) in patients affected by the *HSPB1* pathogenic variant, with a mean of 16 years of illness. 

Clinical and electrophysiological data of *HSPB1* and *MPZ* patients are listed in Table S1.

Based on our findings, we were able to highlight a neuropathy phenotype that differs from the classical ones, whose features can be summarized as follows: (1) the onset of the disease during adulthood (fourth decade on average); (2) the early exclusive or prevalent involvement of the lower limbs; (3) the mild to moderate severity of the disease. Although all CMT neuropathies cause length-dependent damage, the upper limbs are frequently clinically involved [12] in CMT1A [13] or CMT1X [14] and predominantly in some forms, such as neuropathies caused by *GARS* and *BSCL2* pathogenic variants [15]. Nerve conduction studies confirm polyneuropathy in all four limbs.

## 4. Discussion

Genetic testing for CMT involves the sequencing of individual genes addressed by the mode of inheritance, clinical and electrophysiological phenotypes, and data about the prevalence of different genetic subtypes, as well as peculiar genotype–phenotype associations. This approach has been transformed by the advent of NGS, where several disease-associated genes are tested in parallel. Nevertheless, the diagnostic rate of massive parallel sequencing tests described in the literature ranges from 4.6% to 93%, according to the analyzed cohort [16,17,18,19,20,21,22,23,24,25,26,27,28,29,30,31,32,33,34,35,36]. In routine clinical practice, the NGS approach more realistically allows us to reach a genetic diagnosis in 30% of genetically undetermined patients when *PMP22* duplication has been previously ruled out [32].

Our study evaluated the frequency of the genetic subtype of CMT patients in a population from a specialized clinical diagnostic setting in northern Italy. In our cohort, 66% of patients obtained a genetic diagnosis (including 17p11.2 duplication), a diagnostic rate that is comparable with previously described epidemiological studies. In 4% (10 out of 277 index cases), the diagnosis was achieved with an NGS approach. The phenotype distribution showed that 86% of diagnosed patients had demyelinating neuropathy, whereas axonal CMT remained largely undiagnosed. These data confirm that copy number variations in *PMP22* or pathogenic variants in three genes (*GJB1*, *MPZ,* and *MFN2*) are responsible for about 90% of genetically determined neuropathies. This genetic prevalence was similar to the prevalence in Europe and North America [5,37,38,39,40,41,42], whereas it differed from those found in Spain and southern Italy, where *GDAP1* pathogenic variants were more frequent due to the founder effect [43,44]. *SH3TC2* was described as a frequently mutated gene in different papers [17,23,35,44], although it represented less than 1% of our cohort because of the adult age of the patients. The remaining genetically diagnosed cases include pathogenic variants in less common genes.

Interestingly, in our case series, *HSPB1* pathogenic variants were found in 3% of genetically determined neuropathies. A similar prevalence was described in a large cohort of Sicily [45] and Spain [44] and even greater (4.6%) in Japan [29], thus suggesting that the higher prevalence of these pathogenic variants could be more likely attributed to their epidemiological distribution rather than being caused by a specific bias, such as the adult population assessed in our study. *HSPB1* was described as the most common cause of dHMN [31,46], but in our population, it also accounts for 9% of the axonal motor-sensory neuropathies (3 out of 33 axonal sensory-motor neuropathies). *MPZ* pathogenic variants were associated with an axonal phenotype in 42% of patients (8 out of 19 patients). Among patients carrying these variants, almost all (86%; 13 out of 15 patients) presented with adult onset. Frequently, *MPZ* variants associated with adult onset presented electrophysiological findings classified as CMT2, with intermediate or normal mNCV [47,48]. From the clinical data available in our cohort, we were able to establish an association between pathogenic variants in *MPZ* or *HSPB1* and a peculiar phenotype characterized by clinical onset after the third decade, initial exclusive or highly prevalent lower limb involvement, and mild to moderate severity. This phenotype, which is strictly length-dependent, is common in patients carrying *HSPB1* and late-onset *MPZ* pathogenic variants. Houlden et al. [49] described the predominant motor involvement in the lower limbs in *HSPB1* pathogenic variants, whereas a similar involvement, predominantly in the lower limbs, was noticed in *MPZ* pathogenic variants with adult onset by Sanmaneechai et al. [47].

The description of a distinct genotype–phenotype association may seem anachronistic in the era of massive parallel genetic testing through NGS. However, NGS requires time and expertise for data analysis and interpretation, although in cases with definite phenotypes, a gene-by-gene approach might still be effective. Moreover, in a NGS context, detailed phenotypic information can be used to guide the interpretation of molecular results [50]. Finally, NGS panels can explore only a very limited part of the coding genomic DNA, which might represent a significant part of the missing heritability in neurologic diseases as well as CMT [32]. It is also important to note that a significant part of the genome is extremely resistant to single-nucleotide variant (SNV)/small indel calling due to a repetitive sequence, causing poor variant detection in some clinically relevant genes [51]. The contribution of these types of variants in the pathogenesis of neurological diseases is increasingly recognized, as in the case of the identification of the *RFC1* pentanucleotide repeat associated with cerebellar ataxia with neuropathy and vestibular areflexia syndrome (CANVAS) [52] and idiopathic sensory neuropathy [53]. Similarly, pathogenic variants in the *SORD* gene were recently identified as the most common recessive inherited neuropathy [32]. *SORD* was not described previously as a gene involved in hereditary neuropathies due to the inability of NGS analysis to call variants because of the presence of the SORD2P pseudogene. These findings underline the possibility that many novel genes involved in neuromuscular diseases remain to be identified. In general, genetic advances in DNA sequencing technologies have led to a continuous increase in genes related to neuromuscular diseases, and in clinical practice, gene panels must be periodically updated. For this reason, we believe that whole-exome sequencing, followed by filtering for defined genes, could be a valid method [54].

Therefore, in our experience, the diagnosis strategy should be flexible and tuned to the clinical features of the patient in order to select the best molecular approach for each patient. Our study confirms that the collaboration of a multidisciplinary team provides better outcomes for patients [55].

## Supplementary Materials

The following supporting information can be downloaded at: https://www.mdpi.com/article/10.3390/life12030402/s1, Table S1: Clinical and electrophysiological data of *HSPB1* and *MPZ* patients.
